# Efficient generation of hPSC-derived midbrain dopaminergic neurons in a fully defined, scalable, 3D biomaterial platform

**DOI:** 10.1038/srep40573

**Published:** 2017-01-16

**Authors:** Maroof M. Adil, Gonçalo M. C. Rodrigues, Rishikesh U. Kulkarni, Antara T. Rao, Nicole E. Chernavsky, Evan W. Miller, David V. Schaffer

**Affiliations:** 1 Department of Chemical and Biomolecular Engineering, University of California Berkeley, Berkeley, CA, USA; 2 Department of Chemistry, University of California Berkeley, Berkeley, CA, USA.; 3 Department of Molecular and Cell Biology, University of California Berkeley, Berkeley, CA, USA.; 4 Helen Wills Neuroscience Institute, University of California Berkeley, Berkeley, CA, USA.; 5 Department of Bioengineering, University of California Berkeley, Berkeley, CA, USA.

## Abstract

Pluripotent stem cells (PSCs) have major potential as an unlimited source of functional cells for many biomedical applications; however, the development of cell manufacturing systems to enable this promise faces many challenges. For example, there have been major recent advances in the generation of midbrain dopaminergic (mDA) neurons from stem cells for Parkinson’s Disease (PD) therapy; however, production of these cells typically involves undefined components and difficult to scale 2D culture formats. Here, we used a fully defined, 3D, thermoresponsive biomaterial platform to rapidly generate large numbers of action-potential firing mDA neurons after 25 days of differentiation (~40% tyrosine hydroxylase (TH) positive, maturing into 25% cells exhibiting mDA neuron-like spiking behavior). Importantly, mDA neurons generated in 3D exhibited a 30-fold increase in viability upon implantation into rat striatum compared to neurons generated on 2D, consistent with the elevated expression of survival markers FOXA2 and EN1 in 3D. A defined, scalable, and resource-efficient cell culture platform can thus rapidly generate high quality differentiated cells, both neurons and potentially other cell types, with strong potential to accelerate both basic and translational research.

Pluripotent stem cells – with their hallmark capacities for unlimited self-renewal and differentiation into any cell type in the body – are a highly promising resource to address a broad range of biomedical problems, including advancing our understanding of normal development and human disease, enabling the discovery of effective drugs, and developing cell replacement therapies. As a prominent example of the latter, stem cell based regenerative medicine for Parkinson’s disease (PD) – with the goal of replenishing A9 type midbrain dopaminergic (mDA) neurons, the mDA neuronal subtype that resides in the substantia nigra and that is specifically affected in PD – has strong clinical potential to alleviate the motor symptoms of this disease^1^,^2^,^3^. Fortunately, several recent studies have greatly advanced our understanding of mDA neuronal development^1^,^4^, and the accompanying development of 2D culture mDA differentiation protocols is paving the way for clinical translation^1^,^2^.

However, standard 2D culture systems generally face challenges for producing high quality and yields of cells. At a minimum, approximately 100,000 mDA neurons would need to engraft and survive within the striatum for effective disease treatment^5^. With purities of ~15–30% hPSC-derived mDA neurons^1^,^6^,^7^, and only 1–5% of implanted cells surviving as TH+ neurons post-implantation in pre-clinical models^1^,^2^,^3^, generating sufficient numbers of cells to treat the estimated 1 million PD patients in the US alone would be challenging. Even producing the ~10^9^ cells typically needed for an *in vitro* pharmacology, toxicology, or genetic screen is daunting^8^,^9^. Furthermore, current mDA neuron derivation systems entail the use of animal- and human-derived culture components that limit reproducibility and risk pathogen transfer^10^,^11^. To achieve higher capacity cell production, a longstanding approach in cell bioprocess engineering is to “scale up” to three-dimensional (3D) platforms rather than “scale out” to additional 2D surface area. The former offers several potential advantages: a more biomimetic 3D environment for cell culture, the potential for higher cell densities per unit culture volume, and ease of harvesting cells for implantation. Suspension or microcarrier culture offers the potential for scale up; however, human pluripotent stem cells in such cultures can aggregate into large clumps whose interiors undergo necrosis or non-specific differentiation^12^,^13^. Unfortunately, agitation, the most common approach to avoid such aggregation, can result in hydrodynamic shear stress that adversely affects cell growth and differentiation^12^,^14^.

Alternatively, cells can be embedded in a biomaterial for 3D culture. Several important studies have explored materials such as alginate, collagen, and hyaluronic acid for hPSC expansion^15^. However, these particular hydrogels face challenges with limited cell expansion, modest cell densities, undefined culture components, difficult cell harvest, and material properties that change during long term cell culture^12^,^13^,^14^,^16^,^17^,^18^, each of which can hinder hPSC expansion and/or differentiation. New systems are thus needed to realize the potential of 3D biomaterials for hPSC expansion and differentiation^19^. As we recently demonstrated, thermoresponsive materials for hPSC encapsulation can address many of these challenges, and additionally generate early stage mDA neuronal progenitors^20^. However, for a variety of applications including disease modeling, drug screening and cell replacement therapy for Parkinson’s disease, large numbers of region-specific, fate-restricted, post-mitotic mDA neurons are required. It is currently unclear whether differentiation and maturation of delicate, post-mitotic neurons could be efficiently accomplished within a 3D material, as material encapsulation and the accompanying diffusion barriers may impact the activity of differentiation patterning factors and/or affect the subsequent viability and function of mature neurons.

Here, we have adapted effective 2D mDA differentiation protocols^1^,^4^ to develop a biochemically defined, 3D system that can derive mature, electrophysiologically functional, and implantable mDA neurons. Interestingly, through extensive characterization, we observed accelerated neurodevelopment in 3D, high expression levels of mDA markers after 25 days of differentiation, and 25% of 3D-differentiated neurons generating spiking patterns indicative of a functional mDA phenotype. Furthermore, 6 weeks after implantation into the rat striatum, 3D-generated mDA neurons demonstrated a 30-fold increase in survival compared to mDA neurons generated on 2D platforms, consistent with higher expression of survival markers FOXA2 and EN1 in 3D. A 3D thermoresponsive material system therefore offers an efficient and effective approach for rapid generation of functional mDA neurons, in a system compatible with scalable production, to meet diverse needs ranging from regenerative medicine to pharmacology screening.

## Results

### Higher numbers of mDA neurons are generated in 3D, with marker expression profiles indicative of a midbrain fate

**Figure 1:**
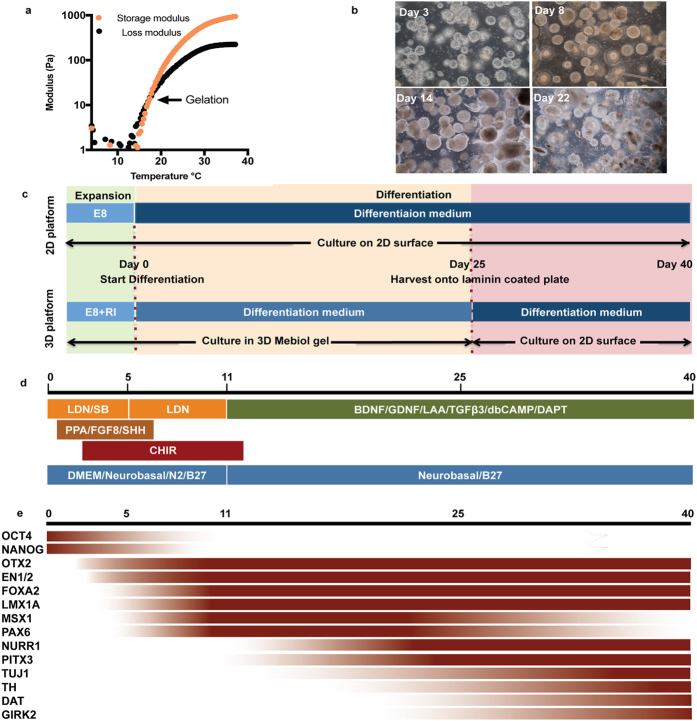
Material properties of 10 w/v % PNIPAAM-PEG are amenable for maintaining pluripotency for hPSCs and generation of hESC derived neurons. (**a**) Rheological measurements of storage and loss moduli of PNIPAAm-PEG gels demonstrating the thermoresponsive liquid to solid transition. Traces are representative of 3 independent experiments. (**b**) Brightfield images of H1 hESC-derived clusters in PNIPAAm-PEG 3D platform at different stages during the mDA differentiation process. Images are representative of n = 4 independent experiments. (**c**) Schematic showing differentiation conditions for 2D and 3D culture. (**d**) Diagram for mDA differentiation protocol. (**e**) Pictorial representation of anticipated expression levels of different markers of interest during mDA neuronal development, based on previously reported trends^25^,^26^,^27^,^28^.

Our early studies demonstrated the importance of material stiffness on stem cell fate, as 2D soft materials substantially promoted both hPSC differentiation into neuroectodermal lineages and adult neural stem cell differentiation into neurons^21^,^22^. Rheological measurements indicated that 10 wt% PNIPAAm-PEG hydrogels had a stiffness of ~1 kPa at 37 °C, a promising range for neuronal differentiation (Fig. 1a)^21^,^22^. After harvest from 2D Matrigel-coated surfaces and at least two passages in 3D, as well as subsequent demonstration of pluripotency maintenance (Figure S1a), mDA neuronal differentiation was induced in 3D (Fig. 1c). For the soluble media components, neural induction was initiated by inhibiting the SMAD pathway (Fig. 1d, orange factors), patterning to a midbrain fate was induced by WNT and SHH signaling pathway activation (Fig. 1d, brown and red factors), and neuronal differentiation was promoted with key neurogenic factors (Fig. 1d, green factors)^1^,^23^. As a parallel control, cells cultured on 2D Matrigel-coated surfaces were differentiated using the same medium conditions, as previously reported^1^,^4^, without further optimization. While 2D hPSC culture typically uses Matrigel^1^,^4^ – a poorly-defined material with a multitude of protein and proteoglycan components^24^ that suffers from lot-to-lot variability and problems with scalability – every component of the differentiation in 3D platform used here was defined. We also note that the gels remained structurally stable and continued to support the differentiating cells over the initial 25-day process (Fig. 1b).

**Figure 2:**
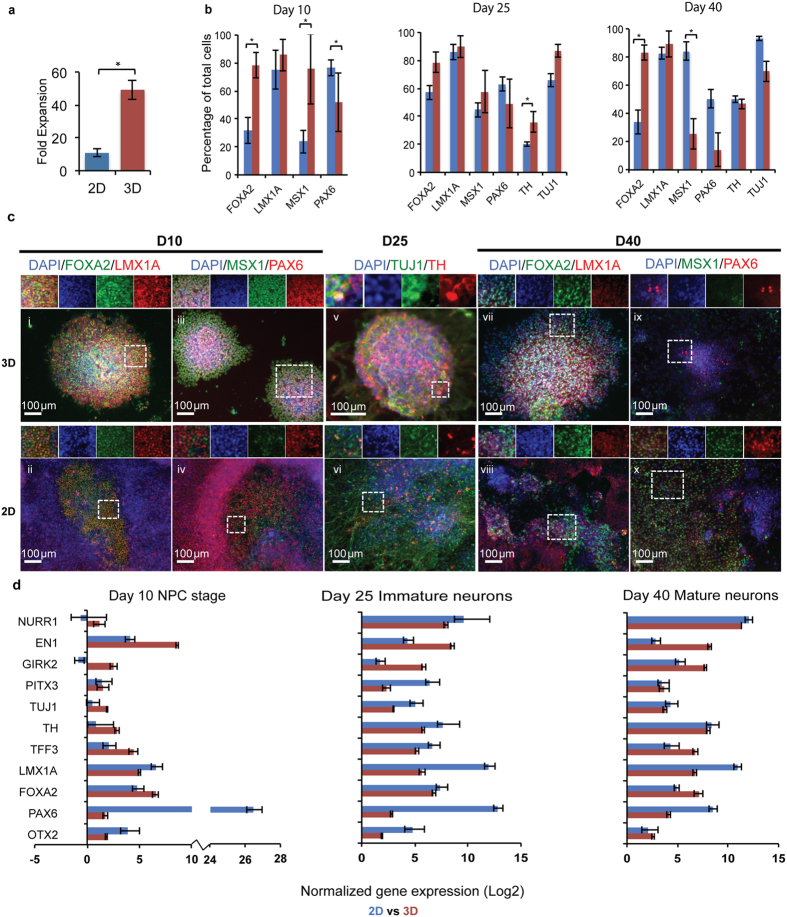
Comparative characterization of H1 hESC derived mDA neurons generated on 2D versus 3D platforms. (**a**) Fold expansion of mDA neurons after 25 days of differentiation in 3D vs. 2D culture. Data are presented as mean ± s.e.m. from n = 3 independent experiments. *p < 0.05 for Student’s t test. (**b**) Quantitative immunocytochemistry comparing mDA marker expression at Days 10, 25, and 40 between 2D (blue) and 3D (red) cultures. Data are presented as mean ± s.e.m. for n = 3 independent experiments. *p < 0.05 for Student’s t test. (**c**) Representative fluorescence images highlighting significant differences between 2D and 3D cultures, corresponding to data presented in (**b**); (i–ii, vii–viii) FOXA2 (green)/LMX1A (red) and (iii–iv, ix–x) MSX1 (green)/PAX6 (red) at Days 10 and 40, and (v–vi) TH (red)/TUJ1 (green) at Day 25. Nuclei are labeled with DAPI (blue). A region of interest (dashed white square) is highlighted along with individual channels for each marker above each image. Scale bars, 100 μm. (**d**) Comparative gene expression analysis at Days 10, 25, and 40 between 2D (blue) and 3D (red) generated mDA neurons. Data are presented as the mean ± standard deviation from triplicates.

Analysis of cell numbers following differentiation indicated that the 3D platform yielded a higher overall number of cells. Specifically, in the standard 2D system, an initial 200,000 cells gave rise to 2 million cells after 25 days of differentiation, for a 10-fold expansion. In contrast, the 3D platform generated 4.9 ± 0.58 million cells from 100,000 starting cells (Fig. 2a), representing a 50-fold expansion. On a volumetric basis, with the same culture media changes in each system (2 mL/day), overall 100,000 cells were generated per mL of medium used in 3D, versus 40,000 cells/ml of medium used on 2D Matrigel-coated surfaces. A higher proliferation rate at the PSC stage^20^, in addition to continued proliferation during the progenitor stage, may have both contributed to the higher yield of cells in 3D.

Differentiation into a mDA neuronal phenotype was investigated first via immunocytochemistry and qPCR analysis at specific time intervals, including at 10 days to investigate mDA progenitor induction consistent with our prior report^20^, but also more importantly at the considerably longer time points of 25 and 40 days to analyze maturation into post-mitotic neurons. In general, cells undergoing neuronal and mDA lineage commitment transition through a defined series of markers^25^,^26^,^27^,^28^, schematically depicted in Fig. 1e. Coexpression of transcription factors FOXA2 and LMX1A denotes a floorplate derived midbrain lineage, previously shown to generate higher quality mDA neurons for PD therapy^1^. Our analysis of marker expression patterns (Figs 2b,c, S2, and S3) showed that 80% of cells expressed LMX1A in both the 2D and 3D platform at each time point. However, FOXA2 was expressed in ~80% of cells in the 3D platform at both the early and late stages of differentiation, 2–3 fold higher than in current 2D control (Fig. 2b). This result demonstrates enhanced development of a floorplate derived midbrain fate in the 3D platform compared to 2D control in this study through the 40 days of differentiation.

High levels of MSX1, an early marker of mDA development, and PAX6, an early neuronal commitment marker, are anticipated at earlier stages of development (Fig. 1e). Interestingly, while a 3-fold higher expression of MSX1 was seen at day 10 in 3D, by day 40 MSX1 as well as PAX6 expression levels were higher in the 2D cultures. Continued PAX6 and MSX1 expression on 2D platforms may indicate slower and/or less extensive differentiation compared to 3D^10^. Furthermore, increased PAX6 expression could also be indicative of an undesirable forebrain fate in our 2D cultures^29^.

In addition to early stage markers, expression of tyrosine hydroxylase (TH), the rate limiting enzyme in dopamine production that is crucial for mDA neuronal function, was significantly higher at day 25 on 3D (36% of cells) than on 2D (20%), suggesting rapid differentiation in 3D. Furthermore, >90% of the TH positive neurons in 3D were also FOXA2 positive (Figure S3c), indicating a floorplate origin. Finally, by D40 of differentiation, TH+ neuronal differentiation plateaued at similar, high levels on both platforms (47% on 3D and 49% on 2D for H1 hESC derived mDA neurons), demonstrating equally strong potential for generating TH+ cells at levels comparable to previous reports^1^,^4^.

To gain deeper insights into mDA differentiation and maturation, qPCR was conducted to quantify several additional markers (Figs 2d and S4). Confirming the immunocytochemistry trends, and in accordance with anticipated marker expression profiles (Fig. 1e), qPCR for mDA neurons generated in both 2D and 3D platforms showed that LMX1A, TH, and Tuj1 levels increased with time or ultimately plateaued between day 25 and day 40 (Fig. 2d). Specific markers of DA maturation – NURR1 and GIRK2 – also increased with time for both platforms. During central nervous system development, mDA neurons arise within a region specified primarily by FGF8 mediated anterior-posterior (via OTX2/GBX2 activity) and SHH mediated dorso-ventral patterning signals^26^. Consistent with natural mDA development, patterning markers specific to this region – including EN1 and OTX2, in addition to FOXA2 and LMX1A – were expressed in mDA neurons generated within both platforms. However, for 2D-generated mDA neurons, expression of the markers OTX2, EN1, and FOXA2 increased, peaked at D25, and then declined. Likewise, PITX3, a potassium channel protein important for mDA neuronal function, and TFF3, a transcription factor specific to the substantia nigra, showed this loss of expression in 2D cultures, contrary to the expected expression profile of developing mDA neurons^25^,^26^,^27^,^28^ (depicted in Fig. 1e). In contrast, these markers increased and then plateaued, with no decrease, in the 3D platform (Figs 2d and S4).

Based on the observed FOXA2 expression patterns, we hypothesized that a desirable ventral fate was established and maintained more effectively in the 3D platform. To investigate this possibility, we examined the expression of additional ventral markers, SHH and CORIN, and found they were established in both platforms by D25, had dropped significantly by D40 in 2D, but were robustly maintained in 3D (Figure S5). In summary, the gene expression patterns obtained here – including FOXA2 and LMX1A (floorplate derived midbrain fate), (ii) TFF3 (substantia nigra specific transcription factor), (iii) PITX3, GIRK2, NURR1, and TH (mature DA markers) – indicate that the cells differentiated in 3D acquired a substantia nigra specific mDA neuronal phenotype faster and in many cases to a greater extent than on 2D, while closely resembling the anticipated trends of mDA development schematically depicted in Fig. 1e^25^,^26^,^27^,^28^.

We also analyzed expression of non-dopaminergic neuronal markers, 5HT for serotonergic and GABA for GABAergic, in mDA neuronal cultures generated in 2D or 3D cultures, and did not find a significant difference (Figure S6). Finally, to demonstrate the general applicability of this platform to generate mDA neurons, we differentiated 3 additional hPSC cell lines – H9 hESCs, WIBR3 hESCs (NIH registry number NIHhESC-1-0079), and 8FLVY6C2 hiPSCs, a cell line derived from healthy fibroblasts^30^ – which all showed robust TH and TUJ1 expression at Day 25 of differentiation (Figure S7).

### mDA neurons generated in 3D are more electrophysiologically active than cells generated in 2D culture

**Figure 3:**
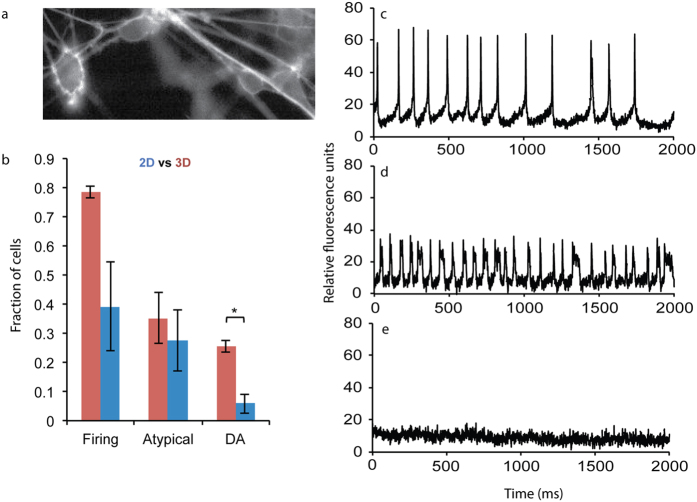
Electrophysiological properties of H1 hESC-derived mDA neurons. (**a**) Representative image of voltage sensitive dye labeled mDA neuron culture. (**b**) Comparative quantification of the fraction of total cell population, from neurons generated on 2D (blue) or in 3D (red) platforms, firing distinct action potentials. Data are presented as mean ± s.e.m from n = 3 independent experiments, for images from 42 total cells in 3D and 48 total cells in 2D. *p < 0.05 for Student’s t test. Representative fluorescence intensity traces corresponding to mDA neuron action potential firing (**c**), atypical firing (**d**), and non-specific noise (**e**).

The capacity to generate action potentials is a hallmark of neuronal maturation and function, and different neuronal phenotypes exhibit distinct, specific firing patterns. A distinguishing feature of A9 type mDA neurons is their spontaneous firing at 2–10Hz^31^,^32^. To date, very few reports have investigated the electrophysiological maturation of hPSC derived mDA neurons, and standard electrophysiology is a low throughput method that only enabled investigation of ~6 neurons per condition^33^. We recently developed a novel approach to optically measure voltage with fluorescent dyes with higher throughput^34^,^35^, and by applying this method to ~100 D40 neurons (culture conditions depicted in Fig. 1c) we observed that 39% of cells generated in 2D exhibited action potentials (Fig. 3), and by contrast 78% of neurons generated in 3D (and subsequently cultured in 2D to facilitate voltage imaging analysis) fired action potentials. Furthermore, 5% of the 2D-generated cells exhibited a firing pattern of periodic spikes at 2–5 Hz^1^, typical of mature A9 type mDA neurons at this stage, compared to 25% of the cells generated on 3D. A previous study, using a similar differentiation technique, also reported very few electrophysiologically active mDA neurons 6 weeks after *in vitro* differentiation on 2D^33^, consistent with our work here. The higher proportion of neurons firing in DA-specific patterns in neurons generated in 3D is consistent with the increased, accelerated expression levels of mature mDA markers (Figs 2 and S4).

### mDA neurons generated in 3D demonstrate increased cell viability, maintain dopaminergic fate, and integrate with host tissue post-implantation *in vivo*

**Figure 4:**
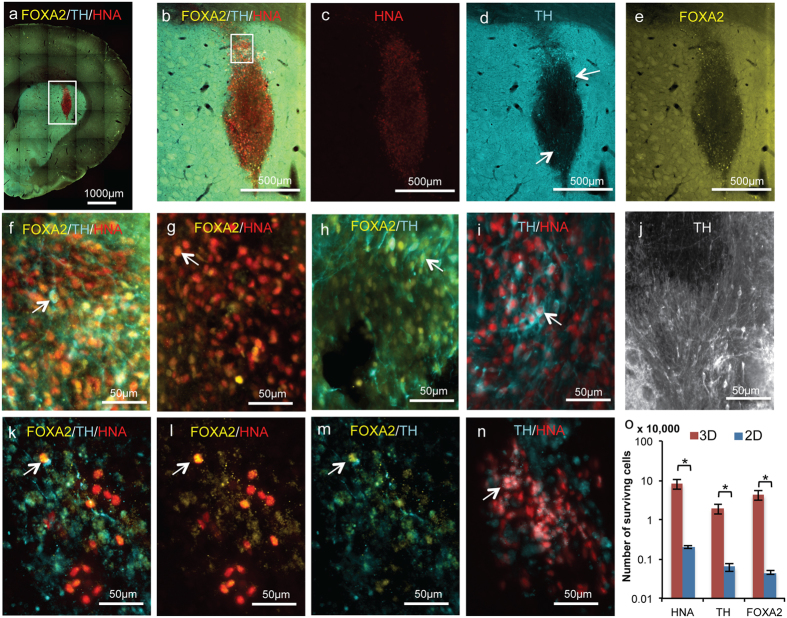
*In vivo* survival of 3D or 2D platform generated mDA neurons in rats. (**a–e**) Graft morphology at 6 weeks post-implantation for mDA neurons generated in 3D, showing expression of HNA, TH, and FOXA2. (**f**) Inset from (**b**), showing coexpression of TH and FOXA2 in surviving HNA+ cells. White arrow shows an example of a cell coexpressing HNA, FOXA2 and TH. Coexpression of FOXA2 and HNA (**g**), of TH and FOXA2 (**h**), and of TH and HNA (**i**), with white arrow showing examples of each. (**j**) TH+ neurite growth within the graft core. (**k–n**) Graft at 6 weeks post-implantation for mDA neurons generated on 2D, showing expression of HNA, TH, and FOXA2. (**k**) Infrequent coexpression of FOXA2 and TH in HNA+ surviving cells (**k**) of FOXA2 and HNA (**l**), and of FOXA2 and TH (**m**), shown by white arrows. (**n**) Coexpression of TH and HNA, shown by white arrow. (**o**) Quantification of total number of HNA+, TH+, and FOXA2+ surviving cells from 4 animals/group for mDA neurons generated in 3D (red bars) or in 2D (blue bars). Data are presented as mean ± s.e.m.

mDA neurons generated in the 3D biomaterial exhibited high quality *in vitro* properties, and to assess their survival and phenotype *in vivo* we implanted 250,000 of these neurons striatally into Fisher 344 rats, a number consistent with prior studies^1^. As a control, we implanted 250,000 mDA neurons that were generated on 2D Matrigel-coated surfaces as previously reported^1^. Six weeks post-implantation, a time point at which previous mDA neuron transplantation studies begin to report functional improvements in PD rat models^36^, we sacrificed the animals and investigated graft survival (Figs 4 and S8). TH and FOXA2 expression was observed in the HNA+ surviving cells among both the 3D (4a–j) and 2D (4k–n) generated mDA neuron groups. Specifically, in the control 2D group, we observed 2020 ± 180 HNA+ cells surviving, corresponding to 0.8% of total cells implanted. Of these HNA+ cells, 31.5% or 638 ± 150 cells were TH+ mDA neurons (Fig. 4o). This is in accord with previous studies that have also reported ~1% survival of unsorted neurons at 6 weeks post-implantation^2^,^3^,^36^.

In contrast, for mDA neurons generated on the 3D platform we noted increased survival of transplanted human cells (HNA positive) and maintenance of the midbrain dopaminergic phenotype (Fig. 4o). Specifically, 82300 ± 20900 HNA+ cells survived, corresponding to 35.6% of implanted cells. Of these, 22.8%, or 18900 ± 4800 cells were TH positive. We therefore observed a substantial 40-fold increase in the total number of cells surviving and a 30-fold increase in the number of TH+ neurons surviving. Furthermore, 22.2% of the surviving HNA+ cells were FOXA2 positive in the 2D controls, whereas 52.6% were FOXA2 positive in the 3D generated mDA neurons. This percentage, in combination with a higher overall survival rate for 3D generated neurons, resulted in 96-fold more FOXA2 positive cells surviving in the 3D grafts compared to the 2D controls. In addition, within the 3D graft, 46.7% of the FOXA2 positive cells were also TH positive, and all of the TH positive cells also expressed FOXA2. The latter result is especially significant as several previous studies have demonstrated the importance of FOXA2 expression in mDA neurons for maintenance of the A9 regional phenotype and overall survival^1^,^37^,^38^.

**Figure 5:**
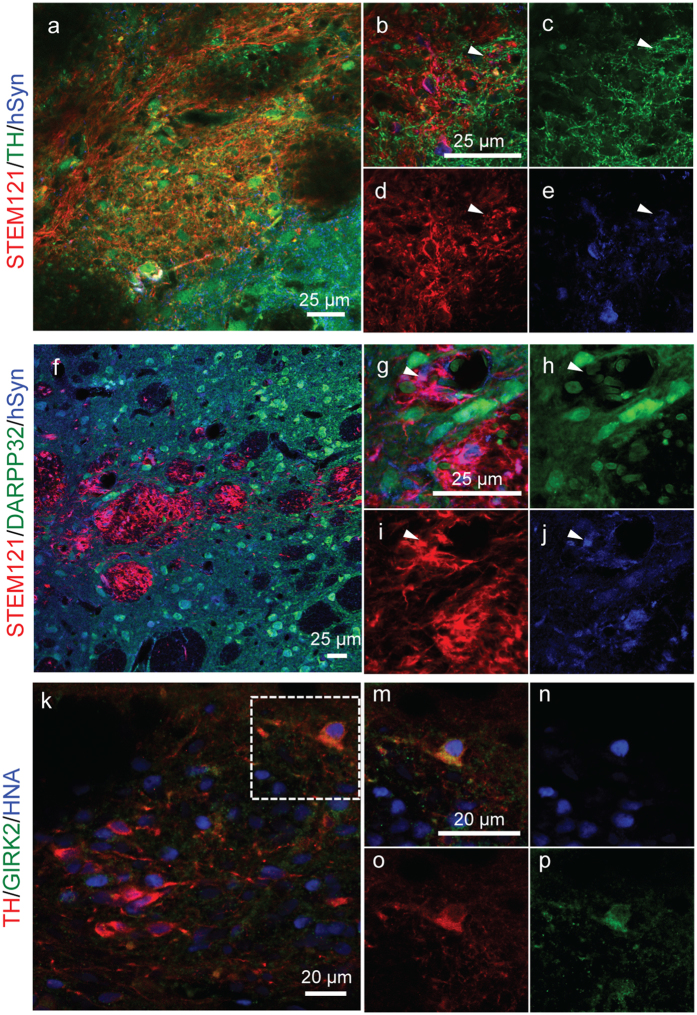
Maturation and synaptic connections formed in 3D generated cell grafts and 6 weeks post-implantation in rats. (**a–e**) Representative image showing coexpression of STEM121 (red), TH (green), and human synaptophysin (hSyn, blue). (**f–j**) Representative image showing STEM121 positive human cells (red) expressing human synaptophysin (hSyn, blue) at the interface with DARPP32+ striatal neurons (green). Images are representative of 4 animals.

Extensive TH positive neurite growth was seen throughout the graft core and graft periphery (Fig. 4j). This observation suggests that the graft had matured and integrated with the surrounding neuronal architecture, which has previously been linked to improved functional recovery^39^. To validate graft maturation and integration with host tissue, we investigated neuronal connectivity – specifically synapse formation as indicated by the expression of the marker synaptophysin – with additional histology (Fig. 5). Human synaptophysin expression was observed throughout the graft among TH+ human neurons (Fig. 5a–e). Furthermore, a hallmark of PD is the loss of connections between TH+ dopaminergic neurons and DARPP32+ striatal neurons, and a criterion of disease-alleviating grafts is to re-generate these connections. Importantly, we observed synapse formation between grafted cells and host DARPP32+ striatal neurons (Fig. 5f–j). Another important hallmark of neuronal maturation *in vivo* is the expression of relevant channel proteins. Accordingly, we observed TH+/GIRK2+ neurons within the graft (Fig. 5k–p). Taken together these results suggest that transplanted cells matured and integrated with the host neuronal architecture, thereby meeting an important criterion for a functional graft.

Finally, we found negligible levels of contaminating serotonergic neurons (5HT+, <0.1%) or astrocytes (GFAP+, <0.6%), and a few GABAergic neurons (GABA+, ~2% for 3D and 10% for 2D) within the grafts for neurons generated in both 3D and on 2D platforms (Figure S9).

## Discussion

There are several important design criteria for a cell culture platform to manufacture functional, clinically relevant cells at large scale: (i) fully-defined, xeno-free culture conditions to enhance reproducibility and scalability, (ii) a scalable culture platform, such as a 3D system, (iii) facile, high viability cell harvesting for passage and implantation, (iv) a microenvironment that supports efficient and effective stem cell differentiation and maturation, and (v) compatibility with long-term culture to enable cell maturation. We show that for hPSC culture and mDA neuronal differentiation, a fully defined, large-scale compatible, thermoresponsive 3D system readily meets each of these criteria.

With soluble media conditions^1^,^4^ adapted to 3D, we engineered a thermoresponsive, synthetic hydrogel system for scalable induction and differentiation of mDA neurons for biomedical applications such as Parkinson’s disease therapy. After 25 days of differentiation, a timepoint previously found to be optimal for cell transplantation in PD models^1^, we report the generation of a ~2-fold higher proportion of mDA neurons (Fig. 2b), and ~5 fold higher numbers of cells generated per volume of medium consumed (Fig. 2a), in the 3D platform compared to a 2D control. A crucial benchmark for clinically relevant mDA neurons is TH expression. The majority *in vitro* differentiation studies report 15–30% TH positive cells after 25–45 days of differentiation on 2D platforms^1^,^6^,^40^,^41^, and in 2D we similarly find 20% TH+ cells at D25. In contrast, in the 3D hydrogel we generated a higher quality, purer mDA neuronal population, with almost double the percentage of TH+ cells (37%) compared to our 2D control. A more enriched population of mDA neurons entails a more efficient use of resources, may increase the therapeutic chances of success, and reduces the risk of side effects from contaminating cell types^42^. Finally, medium (including small molecules and growth factors) is one of the most resource intensive components in cell production, and notably 100,000 neurons were generated per ml of medium used in 3D, compared to 40,000/ml on 2D.

In addition to TH expression, the regional identity of these mDA neurons is important for therapeutic application, as in general DA neurons can be subdivided into several types based on their spatial location, function, and gene expression profiles^26^. In particular, A9 type mDA neurons from the substantia nigra, the neuron type most affected in PD, is most promising in regenerative therapies, while other DA neurons perform suboptimally^43^. Here, we show the continued expression of the important markers FOXA2, EN1, and PITX3 – together known to specifically regulate the development of A9 type mDA neurons^44^ – within cells generated in 3D, whereas in 2D their expression declined significantly after day 25. Moreover, TFF3, a marker highly expressed in the substantia nigra^1^, is expressed at higher levels in 3D at 40 days. Hence, mDA neurons generated in the 3D biomaterial effectively establish and maintain a substantia nigra specific midbrain fate.

Characterization of marker expression alone does not fully reflect the maturity level or functionality of the generated cells. Functional, electrophysiological characterization provides valuable information on neuronal type, quality, and maturity. Prior studies of mDA neuronal development have characterized their firing rates via patch-clamp electrophysiology^45^; however, patch-clamping, the current standard for electrophysiological measurements, has limited throughput that precludes analysis of a large number of cells, with studies typically investigating ~6 neurons per condition^33^. Therefore, the use of voltage imaging allows cellular functional characterization in a higher throughput manner, better representing of the entire population while offering valuable information beyond TH expression. Here, optical electophysiological measurements of nearly 100 total cells showed that a 5-fold higher proportion of 3D-generated exhibited mDA neuron firing patterns compared to 2D-differentiated cells, which correlates with higher expression levels of mature mDA markers, region-specific markers, and mDA survival markers. Thus, in the current study, mDA neuron generation on the 3D platform outperformed 2D, though further optimization could improve 2D performance, and additional 2D vs 3D comparisons using different medium conditions and differentiation protocols may be informative.

Material properties of the PNIPAAm-PEG system may play an important role in supporting the effective generation of a mDA neuronal fate. Importantly, we show that medium conditions optimized for differentiation on 2D platforms may be effectively translated to 3D, and conceivably obstructive diffusion limitations may be overcome with an appropriately permeable biomaterial. Also, material features such as a 3D geometry, stiffness^21^, topography^46^, chemical functionalities^47^, porosity, and degradability^15^ can in general affect stem cell differentiation^19^. While there have been strong advances in our understanding of mechanotransduction^48^, substantial further advances are needed to elucidate the precise molecular mechanisms by which this and other material properties singly or in combination are integrated to regulate cell function, especially in 3D systems. Further experiments to systematically and combinatorially explore culture platform parameters will help elucidate how cells interpret and respond to material properties during differentiation, and thereby offer further opportunities to control cell differentiation and maturation.

Following differentiation, continued expression of both FOXA2 and EN1 is beneficial for the long-term *in vivo* survival of mDA neurons^49^,^50^. FOXA2 is crucial in the early patterning and later maturation of midbrain dopaminergic neurons^51^, and it enhances mDA neuron survival^50^. FOXA2 overexpression can even induce mDA neuronal differentiation from mESCs, and deletion of a single allele of FOXA2 leads to the development of PD in mice^50^. Likewise, EN1 is naturally expressed in all mDA neurons, and EN2 in a small fraction of them. mDA neurons are absent in EN1/EN2 double knockout mice, demonstrating that these factors and in particular EN1 is required for mDA survival^49^. Recently, Kirkeby *et al*. found that EN1+ progenitors transplanted in animal models resulted in grafts rich in DA yield and density^52^. The fact that both FOXA2 and EN1 are robustly maintained in 3D differentiation, but not as effectively in 2D, may indicate that the former are primed for long-term survival. Consistent with this hypothesis, we observed a 40-fold improvement in overall post-implantation survival and a 30-fold improvement of TH+ neuronal survival for mDA neurons generated in the 3D biomaterial compared to those generated on 2D (Fig. 4o), which interestingly correlated to an overall 96-fold increase in the number of FOXA2+ surviving cells produced in 3D. Another potential reason underlying the improved survival of 3D generated cells may be the cell harvest procedure utilized for transplantation. Specifically, the simple, temperature-regulated liquefaction of the encapsulating gel may facilitate higher viability cell harvest compared to mechanical lifting of cells cultured on 2D. Additionally, consistent with the 0.8% post-implantation survival of 2D generated TH+ mDA neurons observed here, previous studies that implanted unsorted mDA neurons generated on 2D surfaces also reported ~1% survival^2^,^3^. The substantial 30-fold increase in TH+ mDA neuron survival may hold promise for improved and accelerated alleviation of PD disease symptoms. Furthermore, all TH positive neurons in the 3D graft coexpressed FOXA2, extensive neurite growth was seen within and surrounding the graft core, and grafted cells formed synaptic connections with surrounding host tissue. The observed high TH+ cell survival can translate to smaller scale cell production systems, enabling more efficient use of resources.

One potential limitation of this biomaterial platform, and 3D culture in general, is the inability to monitor cell morphology in real time. 2D platforms allow easy visualization of cultured cells, which may, to an extent, facilitate convenient, visual monitoring of differentiation outcomes in some instances. However, this potential limitation of 3D platforms may be offset by the rapid, resource efficient, scalable generation of target cell types within a biomimetic, 3D environment. Additionally, reporter cell lines, advanced imaging techniques and identifying characteristic morphology of neuronal clusters may further enable visual monitoring of 3D neuronal cultures.

## Conclusion

Building upon recent advances in mDA differentiation from hPSCs, we have employed a fully defined, thermoresponsive, 3D hydrogel system to generate a ~5-fold higher yield of cells 25 days after differentiation, with a ~5 fold higher proportion of neurons exhibiting functional mDA electrophysiological behavior, compared to cells generated on our 2D controls. Importantly, the cells differentiated in the 3D platform showed temporal marker expression profiles that emulate natural mDA development. Furthermore, high expression of survival markers FOXA2 and EN1 in 3D platforms potentially resulted in a 30-fold increase in survival of TH positive mDA neurons post-implantation *in vivo*. With material properties that support strong neuronal differentiation and maturation, this 3D platform offers efficient, resource effective, and large-scale compatible generation of functional mDA neurons, suitable for applications in drug screening and regenerative medicine. Finally, this general platform technology may prove useful for the production of other neurons, glia, and non-neural cell types to aid the development of cell replacement therapies to treat a range of human disease.

## Materials and Methods

### hESC culture and maintenance

For culture in 2D, H1, WIBR3, H9 hESCs, or 8FLVY6C2 hiPSCs were grown on Matrigel- (Corning, Corning, NY) coated 6 well plates in E8 medium with supplement (Invitrogen, Grand Island, NY) and passaged every 4–5 days. For culture in the 3D platform, hPSC colonies were first grown on Matrigel-coated 2D surfaces for at least 2 passages. Colonies were then harvested with Accutase (Life Technologies, Grand Island, NY) dissociated to single cells, and seeded in Mebiol gels (Cosmobio, Carlsbad, CA) at 1–2000 cells/μl. Cells were maintained in E8 with supplements and 10 μM ROCK inhibitor (Selleckchem, Houston, TX), and passaged with Accutase as single cells every 5 days.

### Dopaminergic differentiation

hPSCs were differentiated to dopaminergic neurons on Matrigel-coated 2D surfaces or within PNIPAAm-PEG 3D gels using a protocol adapted from previously established differentiation techniques^1^,^4^. For 3D, cells harvested from 2D were first adapted to the 3D hydrogel for 2 consecutive single cell passages in supplemented E8 medium with 10 μM ROCK inhibitor. 5 days after the third single cell passage, differentiation was initiated with dual-SMAD inhibition using 100 nM LDN193189 (Stemgent San Diego, CA) and 10 μM SB431542 (Selleckchem, Carlsbad, CA). Media conditions were maintained throughout differentiation as depicted in Fig. 1c, with small molecule and protein concentrations as previously described^1^,^4^. N2 (Life Technologies, Grand Island, NY), B27 (Life Technologies, Grand Island, NY), Glutamax (Invitrogen, Grand Island, NY), 100 ng/ml FGF8 (Peprotech, Rocky Hill, NJ), 3 μM CHIR99021 (Stemgent, San Diego, CA), 20 ng/ml BDNF (Peprotech, Rocky Hill, NJ), 20 ng/ml GDNF (Peprotech, Rocky Hill, NJ), 2 μM Purmorphamine (Stemgent, San Diego, CA), 0.5 mM DibutyrylcAMP (Santa Cruz Biotechnologies, Dallas, TX), 10 μM DAPT (Selleckchem, Carlsbad, CA), 1 ng/ml TGFβ3 (R&D Systems, Minneapolis, MN) and 0.2 mM L-Ascorbic Acid (Sigma-Aldrich, St Louis, MO) were used in media formulations as needed.

### Action potential analysis by voltage sensitive dyes

Day 25 mDA neurons were harvested from 3D gels or 2D, seeded as clusters on laminin-coated 12 mm glass coverslips, and cultured for 15 days using differentiation medium as described above. Voltage sensitive dyes were then used to monitor the electrophysiological activity of mDA neurons using previously reported methods^34^,^35^,^53^. For experiments measuring spontaneous neuronal activity, the cells were incubated with voltage sensitive dye (1 μM) in HBSS at 37 °C for 15 min. The dye was excited using a 510 nm LED and images were acquired with a W-Plan-Apo 63x/1.0 objective (Zeiss) and OrcaFlash4.0 sCMOS camera (sCMOS, Hamamatsu). For image processing, regions of interest encompassing cell bodies were drawn in ImageJ, and the mean fluorescence intensity across the video was extracted. These traces were then bleach corrected in Clampfit 10 (Molecular Devices), and action potentials were detected using a threshold search using a value of 3x the standard deviation of the baseline fluorescence in each trace. Cells firing at a rate between 2 and 5 Hertz were classified as possessing the mDA stereotypical activity^1^, while cells with firing rates outside of this range were designated as “atypical”.

### *In vivo* transplantation and immunohistochemistry

All stem cell procedures and procedures in animals were performed following NIH guidelines for animal care and use and were approved by the UC Berkeley Animal Care and Use Committee (ACUC), the Committee for Laboratory and Environmental Biosafety (CLEB), and the Stem Cell Research Oversight committee (SCRO).

Day 25 mDA neurons differentiated in parallel on 2D Matrigel coated surfaces or in 3D biomaterial platforms, one batch for each platform, were harvested and dissociated to small ~50–100 μm clusters using 0.5 mM EDTA and pipetting. 250,000 cells were implanted into the striatum of isoflurane anesthetized 150–200 g adult female Fischer 344 rats (at stereotaxic coordinates AP: +1.0, ML: −2.5, DV −5.0). Four animals were assigned per group. 10 mg/kg Cyclosporine was injected intraperitoneally daily starting 24 h before surgeries and until the animals were euthanized. 6 weeks after cell implantations, animals were transcardially perfused with 4% PFA. Brains were harvested and incubated in 4% PFA overnight, and transferred into a 30% (w/v) sucrose solution the following day.

After sufficient dehydration, brains were sliced into 40 μm sections using a microtome. Primary antibodies diluted in primary blocking buffer (5% donkey serum, 2% BSA, 0.1% Triton x100) were incubated with the brain sections for 72 h with gentle rocking at 4 °C. Following incubation, brain sections were rinsed once with 0.2% Triton in PBS and washed three times with 0.1% Triton in PBS, followed by a 4 h incubation with appropriate secondary antibodies diluted in 2% BSA in PBS. DAPI was added 30 min before the end of secondary antibody incubation period. Brain sections were subsequently washed with PBS and mounted. A Zeiss Axioscan Z1 automated slide scanner and a Zeiss AxioObserver fluorescent microscope was used for imaging, and Zen 2.0 software was used for analysis.

The percentage of cell survival was quantified using the cell counter feature on ImageJ, following Abercrombie’s method as previously described(Abercrombie, 1946). All cells positive for HNA and TH were counted from zoomed-in pictures originally acquired at 5x magnification on the Zeiss Axioscan slide scanner, of every 5^th^ brain section spanning the injection site (~8 sections across ~50 total sections). The total number of HNA and TH positive cells were then extrapolated from these counts. Furthermore, all HNA positive cells were counted from three representative sections for each rat brain, and imaged at 20x magnification on the Zeiss AxiObserver. Cells double positive for TH/HNA and FOXA2/HNA were then quantified in these images.

## Additional Information

**How to cite this article**: Adil, M. M. *et al*. Efficient generation of hPSC-derived midbrain dopaminergic neurons in a fully defined, scalable, 3D biomaterial platform. *Sci. Rep.* **7**, 40573; doi: 10.1038/srep40573 (2017).

**Publisher's note:** Springer Nature remains neutral with regard to jurisdictional claims in published maps and institutional affiliations.

## Supplementary Material

Supplementary Information
