# Supplementary material for: Efficient generation of hPSC-derived midbrain dopaminergic neurons in a fully defined, scalable, 3D biomaterial platform
Source: Sci Rep. 2017 Jan 16;7:40573. doi: 10.1038/srep40573 (PMC5238378; doi:10.1038/srep40573)
Supplement: Supplementary Information [file srep40573-s1.pdf]

Supplemental Information

Efficient generation of hPSC-derived midbrain dopaminergic neurons in a fully defined, scalable, 3D biomaterial platform

Maroof M. Adil[1], Gonçalo M. C. Rodrigues[1], Rishikesh U. Kulkarni[2], Antara T. Rao[3], Nicole E. Chernavsky[4], Evan W. Miller[2], David V. Schaffer[1,3,4]

[1]Department of Chemical and Biomolecular Engineering, University of California Berkeley, Berkeley, CA, USA; [2]Department of Chemistry, University of California Berkeley, Berkeley, CA, USA; [3]Department of Molecular and Cell Biology, University of California Berkeley, Berkeley, CA, USA; [4]Department of Bioengineering, University of California Berkeley, Berkeley, CA, USA

Materials and Methods

Rheology

Using an Anton Paar rheometer (Ashland, VA), storage (E') and loss (E") moduli of Mebiol were measured under constant strain of 1 % at 1 Hz from 4 °C to 37 °C with a temperature increase rate of 2 °C/min. Rheoplus software was used to analyze the results.

Quantitative Immunocytochemistry

Cell clusters in the 3D platform were harvested by dissolving the gel in cold PBS, centrifuging, and resuspending in EDTA. Cells grown on 2D surfaces were harvested using EDTA. After pipet mixing and breaking clusters up to appropriate sizes (~100μm),

the smaller clusters were reseeded onto a 0.01 % poly-L-ornithine (Sigma, St. Louis, MO) plus 20 µg/ml laminin (Invitrogen, Grand Island, NY)) coated plate for 1 day in the relevant differentiation medium before fixing with 4 % paraformaldehyde. Following PBS washing, cells were blocked for 1 h at room temperature on a rocker with primary blocking buffer (2% BSA, 5% donkey serum, 0.3 % Triton X 100 in PBS). Primary antibodies diluted in primary blocking buffer were incubated with the cells overnight on a rocker at 4 °C. The next day, cells were rinsed once with 0.2 % Triton in PBS and washed three times with 0.1% Triton in PBS, followed by a 2h incubation with appropriate secondary antibodies diluted in 2 % BSA in PBS. DAPI was added 30 min before the end of secondary antibody incubation period. Cells were subsequently washed three times with PBS and imaged on a Zeiss AxioObserver fluorescent microscope. The various primary and secondary antibodies used, and their respective dilutions, are presented in Supplementary Table 1. For the transcription factors FOXA2, LMX1A, MSX1, PAX6, OCT4, and NANOG, the number of cells labeled positive was counted in Cell Profiler and expressed as a percentage of total DAPI labeled cells in the image. Percentage of cells positive for neuronal markers Tuj1 and TH were manually counted using the cell counter feature in ImageJ. For each marker, differentiating cells were imaged from three different biological replicates, and >6 representative images were taken for each marker set. On average, ~2500 nuclei were counted per image and percentage positive for markers of interest quantified.

qPCR

At specified intervals during differentiation, cells were harvested, and mRNA was extracted using a Qiagen RNA extraction kit (Qiagen) according to the manufacturer's

instructions. mRNA was reverse-transcribed using iScript reverse transcriptase (Bio-Rad) and quantified on an iQ5 RT-PCR detection system (Bio-Rad). Data were normalized to GAPDH expression and analyzed using the $2^{\Delta\Delta Ct}$ method. The primers used for qPCR are presented in Supplementary Table 2.

Statistical analysis

Statistical significance was calculated for n≥3 biological replicates with Student's t -Test assuming a two-tailed homoscedastic distribution.

References

Abercrombie, M., 1946. Estimation of nuclear populatin from microtome sections. Anat. Rec. 94, 239–247.

Antibodies	Company	Cat. No.	Host	Dilution
FOXA2	Santa Cruz	sc-101060	Mouse	1:500
LMX1A	Millipore	MAB10533	Rabbit	1:500
TUJ1	Invitrogen	480011	Mouse	1:500
TH	Pel-freeze	P40101	Rabbit	1:500
OCT4	Santa Cruz	sc-5279	Mouse	1:200
NANOG	Santa Cruz	sc-33759	Rabbit	1:200
MSX1	Hybridoma bank	4G1-C	Mouse	1:100
PAX6	Biolegend	PRB-278P	Rabbit	1:300
TH	Abcam	ab76442	Chicken	1:1000
HNA	Millipore	MAB1281	Mouse	1:250
hSyn	R&D Systems	AF5555	Mouse	1:50
DARPP32	Abcam	ab40801	Rabbit	1:100

5-HT	Immunostar	20080	Rabbit	1:10000
Alexa 647 Donkey anti Ms	Jackson Immunoresearch	A31571	Donkey	1:1000
Alexa 555 Donkey anti Rb	Jackson Immunoresearch	A31572	Donkey	1:1000
Alexa 488 Donkey anti Ch	Jackson Immunoresearch	703-545-155	Donkey	1:1000

Table S1. Antibodies used in immunocytochemistry

**Gene**	**Forward primer (F)**	**Reverse primer (R)**	**Amplic on size**	**Tm F; Tm R**
OCT4	CACCATCTGTCGCTTCGAGG	AGGGTCTCCGATTTGCATATCT	132	62.6; 60.7
NANOG	AAGGTCCCGGTCAAGAAACAG	CTTCTGCGTCACACCATTGC	237	62; 61.9
PAX6	AACGATAACATACCAAGCGTGT	GGTCTGCCCGTTCAACATC	120	60; 60.8
FOXA2	GGAGCAGCTACTATGCAGAGC	CGTGTTCATGCCGTTCATCC	83	62.3; 61.7
OTX2	CATGCAGAGGTCCTATCCCAT	AAGCTGGGGACTGATTGAGAT	200	60.8; 60.6
PITX3	CCTACGAGGAGGTGTACCCC	CCCACGTTGACCGAGTTGA	112	62.6; 61.9
DAT	TTTCTCCTGTCCGTCATTGGC	AGCCCACACCTTTCAGTATGG	223	62.4; 61.8
TH	GGGCTGTGTAAGCAGAACG	AAGGCCCGAATCTCAGGCT	107	60.7; 63
NURR1	ACCACTCTTCGGGAGAATACA	GGCATTTGGTACAAGCAAGGT	175	60; 61.1
GIRK2	CACATCAGCCGAGATCGGAC	GGTAGCGATAGGTCTCCCTCA	103	60.3; 61.2
TUJ1	GGCCAAGGGTCACTACACG	GCAGTCGCAGTTTTCACACTC	85	62.3; 62
LMX1A	ACGTCCGAGAACCATCTTGAC	CACCACCGTTTGTCTGAGC	248	61.8; 61
EN1	GAGCGCAGGGCACCAAATA	AATAACGTGTGCAGTACACCC	138	62.7; 60.3
SHH	CTCGCTGCTGGTATGCTCG	ATCGCTCGGAGTTTCTGGAGA	176	62.8; 62.7
CORIN	CCTCCTCCGGTTCCTATTGC	CCAAAGGTTCACTCCCATTTGA	131	61.7; 60.4
GAPDH	GGAGCGAGATCCCTCCAAAT	GGCTGTTGTCATACTTCTCATGG	197	61.6; 60.9

Table S2. Primers used in qPCR

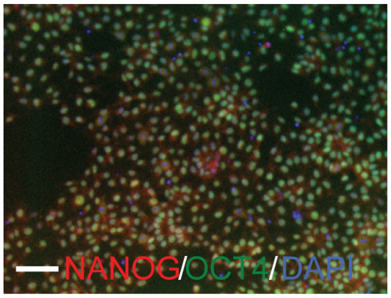

Figure S1. Immunocytochemistry for pluripotency markers OCT4 (green) and NANOG red) in H1 hESCs maintained in 3D culture prior to differentiation. Nuclei are labeled with DAPI (blue). Images are representative of n=3 independent experiments. Scale bar, 100μm.

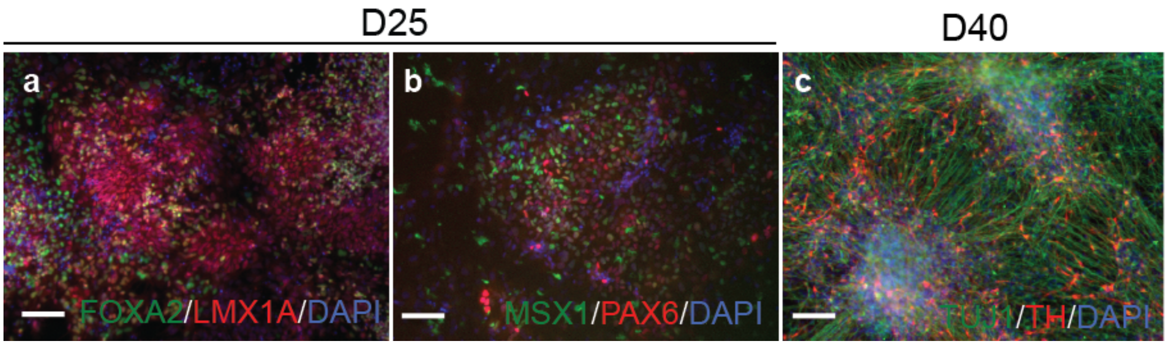

Figure S2. Immunocytochemistry for H1 hESC derived mDA neurons generated on 2D. Representative images (for n =3 independent experiments) showing a) FOXA2 (green)/LMX1A (red) and b) MSX1 (green)/PAX6 (red) at Day 25, and c) TH (red)/TUJ1 (green) at Day 40. Nuclei are labeled with DAPI (blue). Scale bars, 100μm.

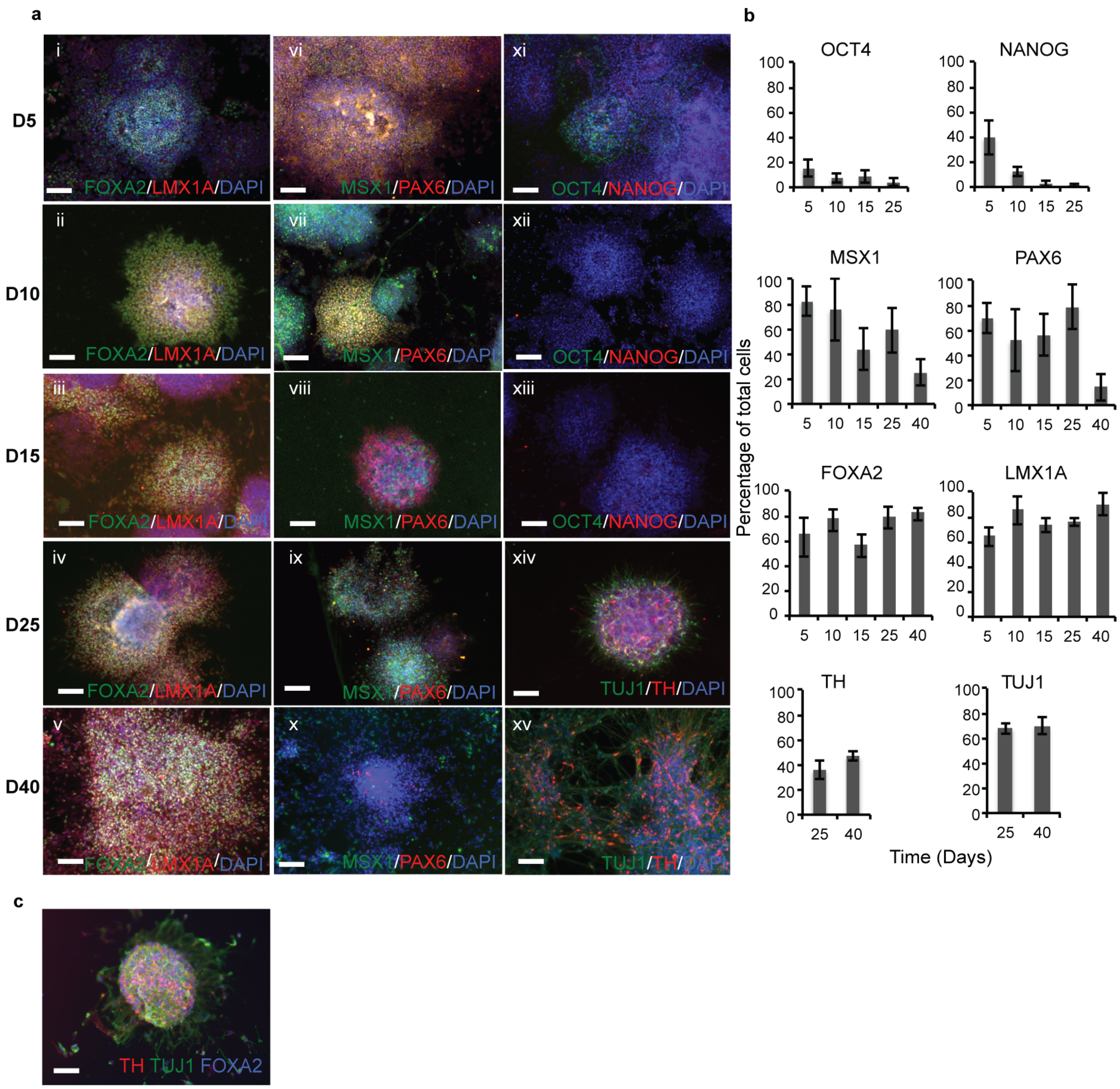

Figure S3: Immunocytochemistry for H1 hESC derived mDA neurons generated on 3D PNIPAAM-PEG gels. a) Immunocytochemistry at Days 5, 10, 15, 25, and 40 for (i-v) FOXA2 (green)/LMX1A (red) and (vi-x) MSX1 (green)/PAX6 (red); at Days 5, 10, 15 for OCT (xi-xiii) (green)/NANOG (red); and at Days 25 and 40 for (xiv and xv) TH (red)/Tuj1 (green). Nuclei are labeled with DAPI (blue) in all images. Images are representative of n=3 independent experiments. Scale bars, 100µm. b) Quantification of data presented in a; data are presented as mean ± s.e.m. for n= 3 independent

experiments. c) Representative image (for n =3 independent experiments) showing TH (red), TUJ1 (green) and FOXA2 (blue) for mDA neurons derived from H1 hESC in 3D. Scale bar, 100μm.

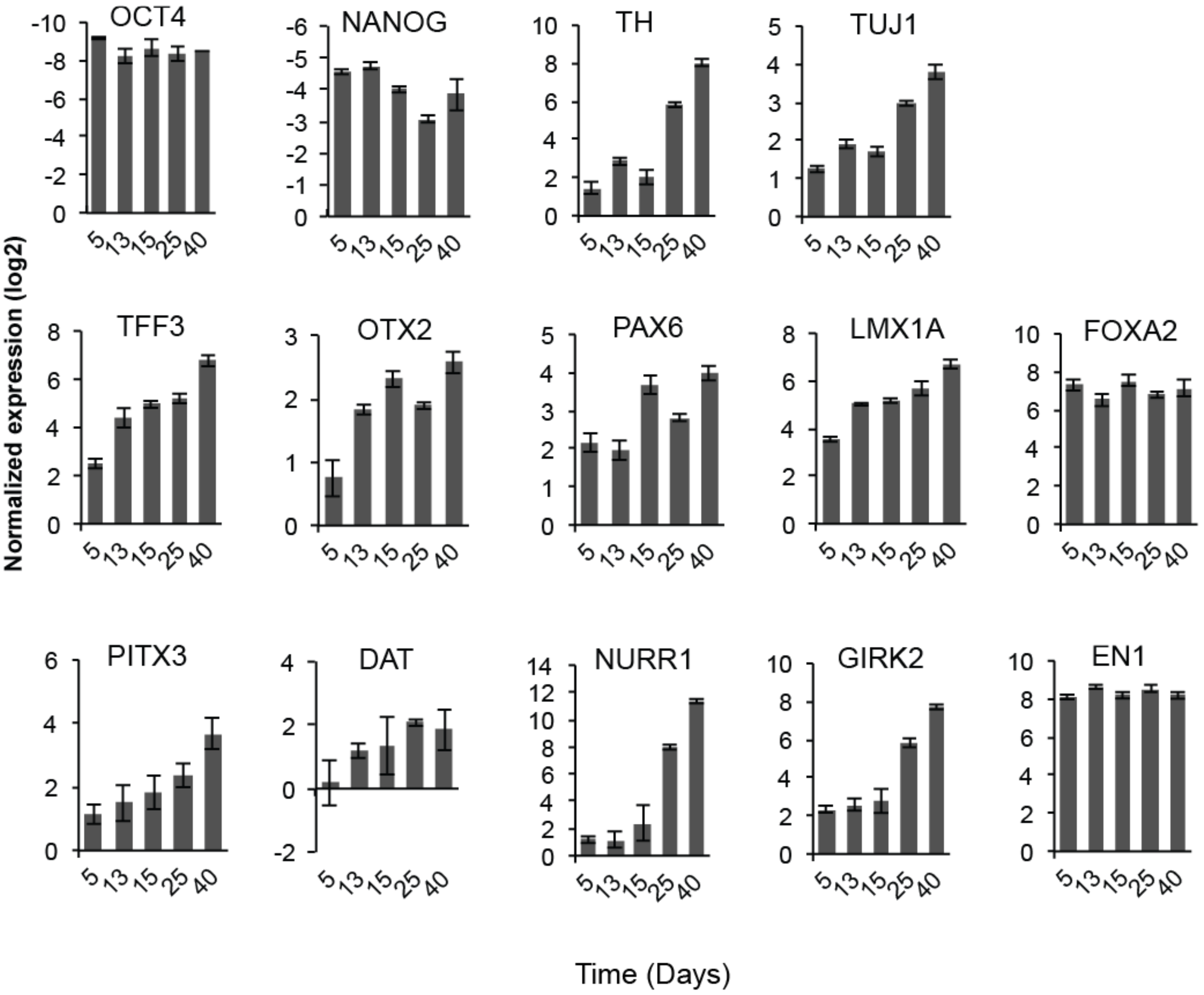

Figure S4: Gene expression analysis of H1 hESC derived mDA neurons generated in 3D PNIPAAM-PEG gels. Data are presented as mean ± standard deviation from triplicates.

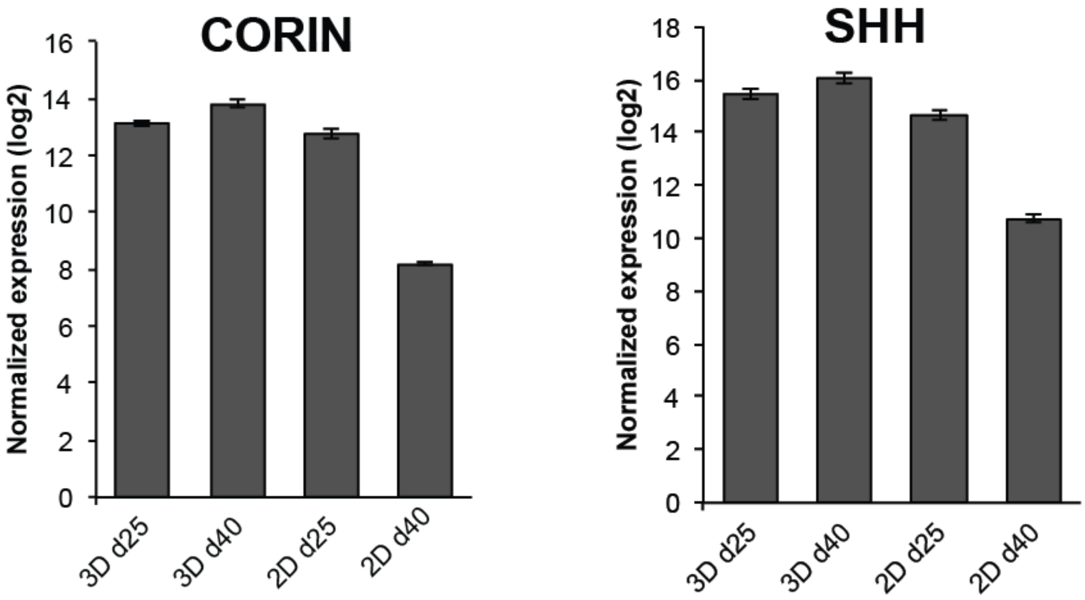

Figure S5. Expression of ventral markers CORIN and SHH in H1 hESC derived mDA neurons generated on 2D or 3D platforms. Data are presented as mean ± s.e.m (triplicates).

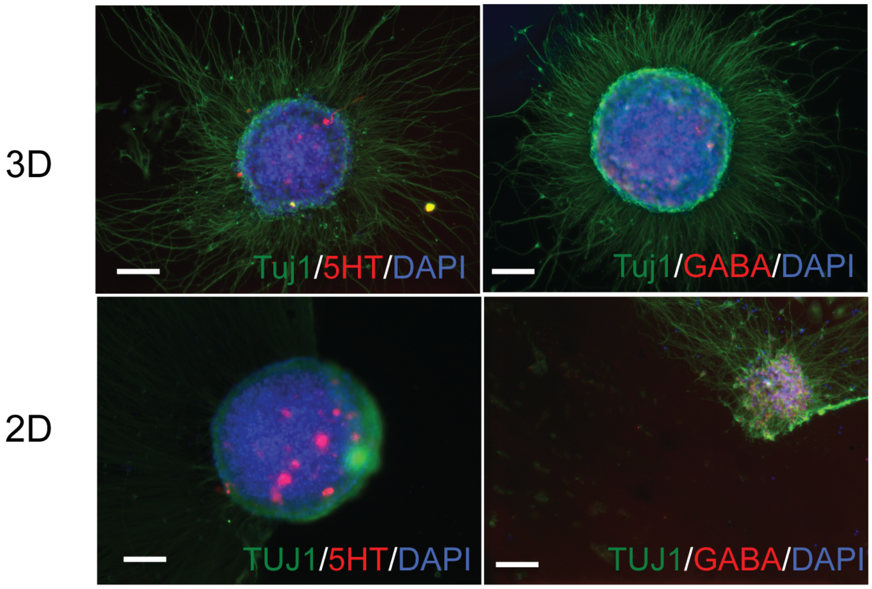

Figure S6. Differentiation directed towards the mDA phenotype yields low levels of serotonergic and GABAergic neurons. Immunocytochemistry at D25 for mDA

neurons generated on 3D (top) and 2D (bottom) showing (left panels) 5HT(red)/TUJ1 (green) and (right panels) GABA (red)/TUJ1 (green). Nuclei are labeled with DAPI (blue). Images are representative of n = 3 technical replicates. Scale bars, 100μm.

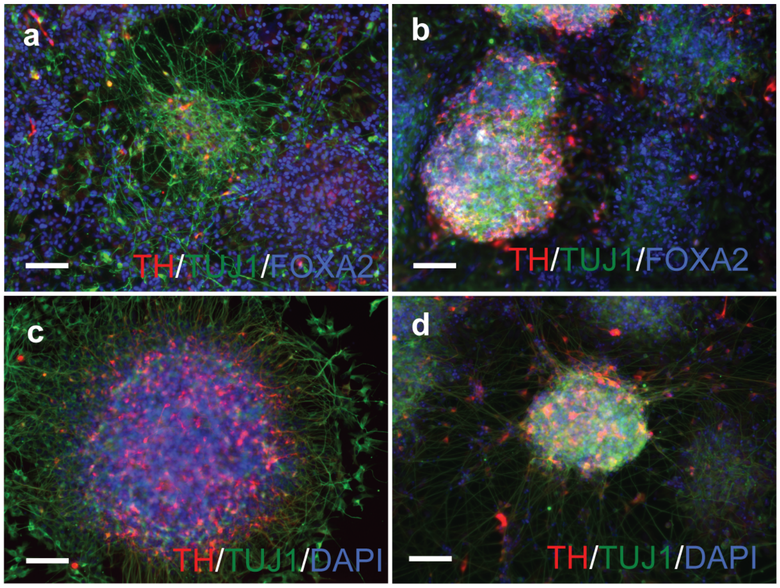

Figure S7.Additional immunocytochemistry for Day 25 mDA neurons derived from various hPSC lines. Representative images (for n =3 independent experiments) showing TH (red), TUJ1 (green) and FOXA2 or DAPI (blue) for mDA neurons derived from a) WIBR3 hESCs on 2D, b) WIBR3 hESCs in 3D, c) H9 hESCs in 3D, and d) 8FLVY6C2 hiPSCs in 3D. Scale bars, 100μm.

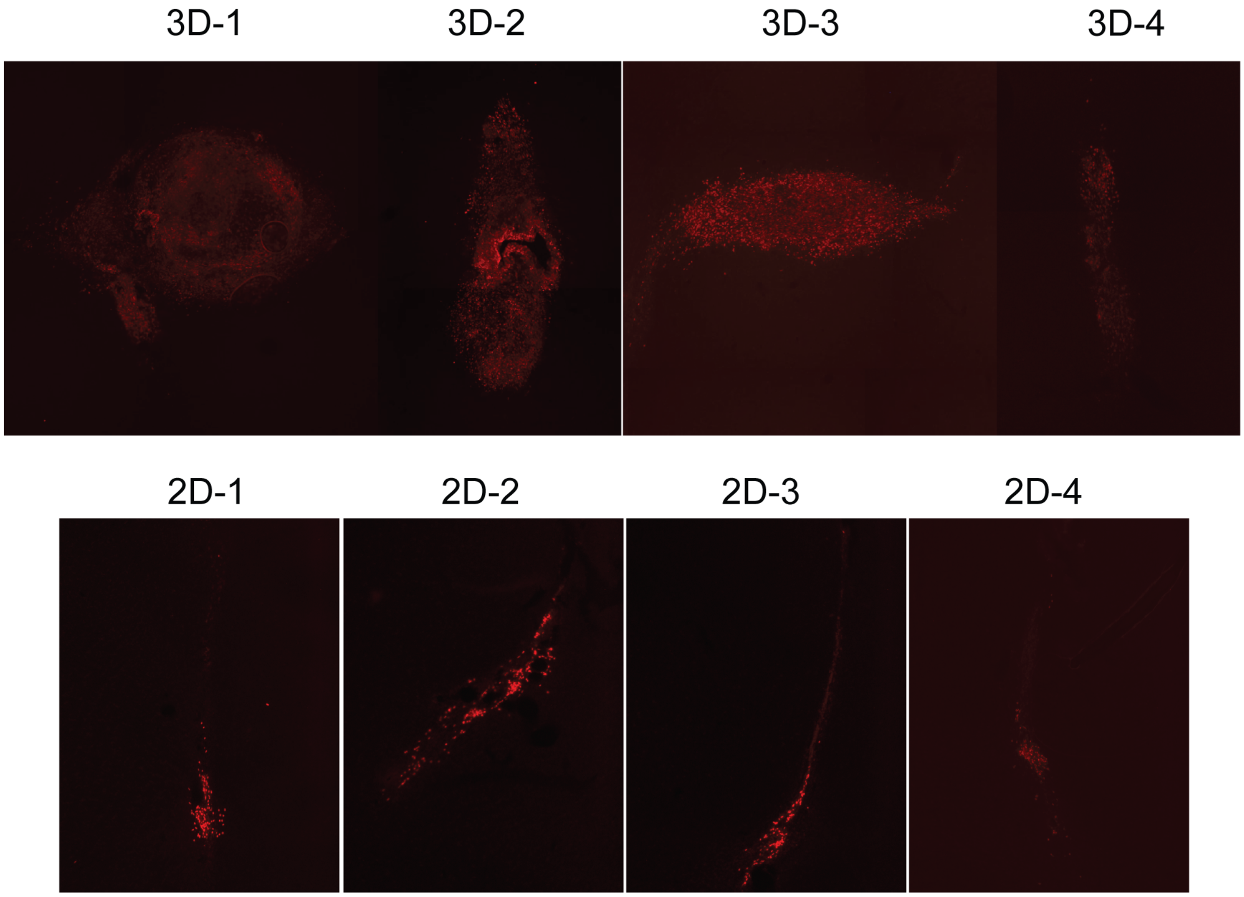

Figure S8. Representative images showing *in vivo* grafts (HNA, red) of mDA neurons generated in 3D (top panel) or on 2D (bottom panel) platforms 6 weeks post-implantation in rats, for each of the animals in the study.

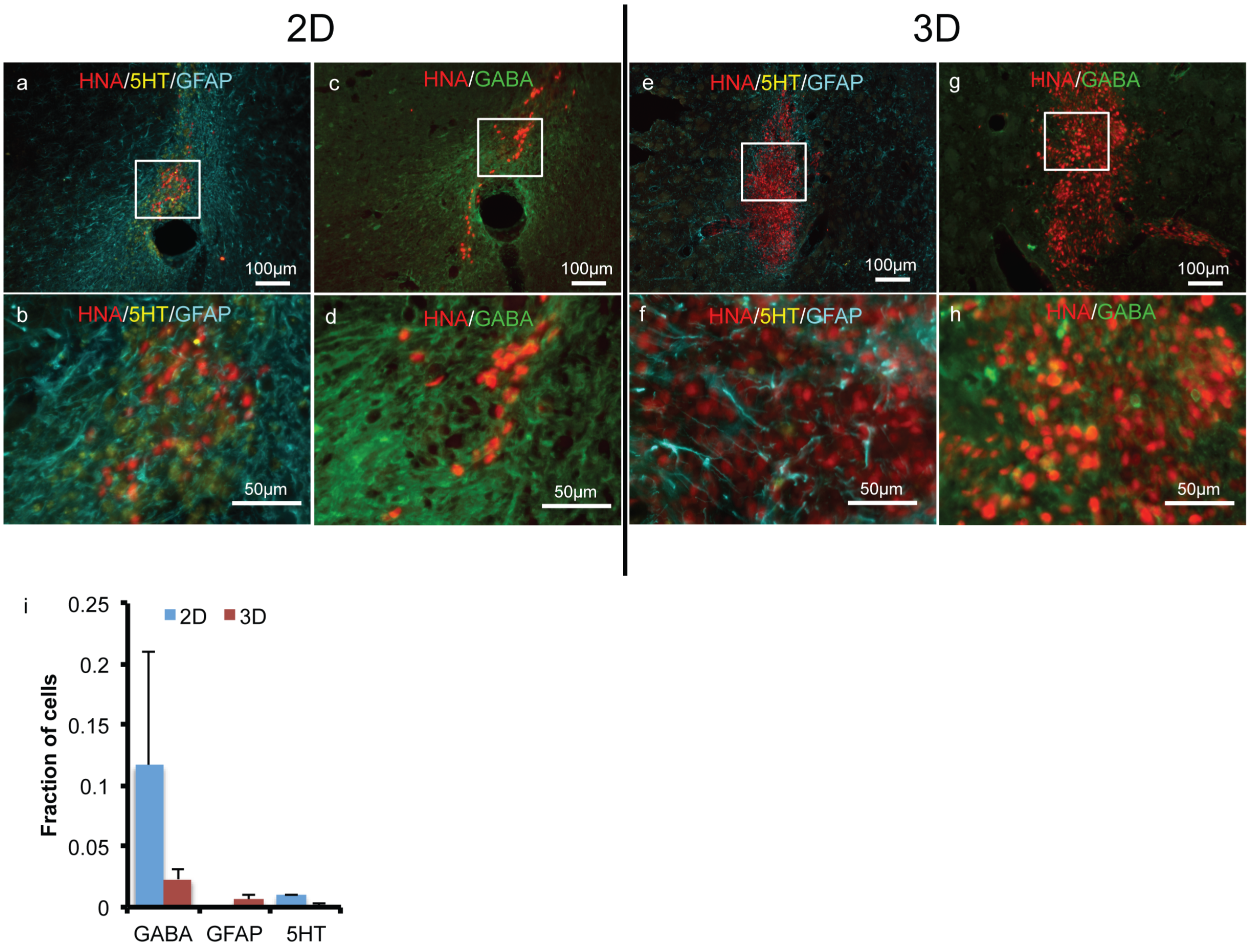

Figure S9. Representative images showing expression of serotonergic, GABAergic and astrocytic markers within *in vivo* grafts of mDA neurons generated in 3D or on 2D platforms 6 weeks post-implantation in rats. (a,b) Serotonergic neurons (5HT+, yellow) and astrocytes (GFAP+, cyan) cells amongst grafted HNA+ (red) cells for mDA neurons generated on 2D (a,b) or in 3D (e,f) platforms. GABAergic neurons (GABA+, green) among grafted HNA+ (red) cells for mDA neurons generated on 2D (c,d) or in 3D (g,h) platforms. Images are representative of 4 rats per group.
